# Clinicopathological and Endoscopic Features of Diffuse Alimentary Lymphoma in 18 Dogs

**DOI:** 10.3390/vetsci12080751

**Published:** 2025-08-12

**Authors:** Florian Bedel, Adeline Betting, Maud Girod, Thomas Chavalle, Daniela Prata, Patrick Lecoindre, Alexis Lecoindre

**Affiliations:** 1 Department of Internal Medicine, Centre Hospitalier Veterinaire OnlyVet, 69800 Saint-Priest, France; 2 Department of Oncology, Centre Hospitalier Veterinaire OnlyVet, 69800 Saint-Priest, France; 3 Department of Anatomopathology, IDEXX Laboratories, 93200 Saint Denis, France

**Keywords:** endoscopy, lymphoma, gastrointestinal

## Abstract

Diffuse alimentary lymphoma (AL) in dogs is an under-characterized entity lacking well-defined diagnostic criteria. This retrospective study evaluated the clinical, endoscopic, histopathological, and immunohistochemical features of 18 dogs diagnosed with diffuse AL between 2017 and 2024. The inclusion criteria for dogs were an abdominal ultrasound, gastrointestinal endoscopy with multiple mucosal biopsies, and the availability of both histopathological as well as immunohistochemical data. Dogs with an intestinal mass were excluded. The duodenum was the most frequently affected segment, with a “cobblestone” endoscopic appearance noted in 53% (9/17) of dogs undergoing upper gastrointestinal endoscopy. Compared to dogs without this feature, those with a “cobblestone” appearance of the duodenal mucosa had significantly lower plasma albumin concentrations (mean: 18.8 g/L, SD: 4.32, range: 19–31 vs. mean: 25.3 g/L, SD: 4.3, range: 19–31; *p* = 0.007), higher CCECAI scores (mean: 11.1, SD: 1.45, range: 9–13 vs. mean: 8.0, SD: 2.27, range: 5–12; *p* = 0.004), and shorter survival time (median: 9 days, range: 4–58 vs. median: 92 days, range: 12–350; log-rank test: *p* = 0.02). While certain endoscopic features—such as a “cobblestone” duodenal mucosal appearance—were associated with more severe clinical and biological profiles, the diagnostic value of these lesions remains to be clarified.

## Simple Summary

Diffuse alimentary lymphoma (AL) is a rare form of intestinal lymphoma in dogs, with limited data available regarding its presentation. In this retrospective study, 18 dogs diagnosed with diffuse AL were analyzed to describe their clinical, endoscopic, and histopathological features. All dogs presented with chronic digestive signs. The most frequent endoscopic abnormality was altered granularity of the duodenal mucosa, with a distinct “cobblestone” appearance, observed in 53% of cases. This feature was significantly correlated with lower albumin concentrations and higher Canine Chronic Enteropathy Clinical Activity Index (CCECAI) scores. The vast majority of lymphomas were of a T-cell phenotype (16/18; 89%), and the overall prognosis was poor (median survival time: 30.5 days), especially in dogs showing a “cobblestone” pattern (median survival time: 9 days). None of the dogs received maximum-tolerated-dose chemotherapy protocols. These results suggest that diffuse AL should be suspected in dogs with severe chronic enteropathy, even in the absence of visible masses upon imaging. Endoscopic assessment, particularly in the duodenum, may reveal mucosal abnormalities that are associated with disease severity, but their diagnostic specificity remains to be determined.

## 1. Introduction

Alimentary lymphoma (AL) is well documented in cats, but studies on dogs remain scarce. AL is one of the most prevalent forms of extranodal lymphoma in dogs, accounting for 5 to 7% of all canine lymphoma cases [1]. According to previous studies, most canine intestinal lymphomas are of a T-cell origin [2,3,4], with high-grade AL the most frequently described, and only a few cases of low-grade AL [2]. The majority of studies describe histological and immunohistochemical aspects [3,5,6,7,8], but pay less attention to clinical features, endoscopic findings, and prognosis of dogs with AL that do not present a well-defined mass.

In human medicine, gastroenterologists aim to standardize the description of endoscopic lesions observed in gastrointestinal lymphomas by using endoscopic terminology that has been consistently reported across multiple publications [9,10]. Although no endoscopic lesion is pathognomonic of the AL type, some lesions are known to be significantly associated with aggressive non-Hodgkin’s lymphoma [10]. In veterinary medicine, no such criteria exist yet.

Endoscopic biopsies are a suitable sampling method for diagnosing AL, particularly because approximately 75% of these lymphomas exhibit epitheliotropism [2]. Endoscopic gastrointestinal biopsies offer several clinical advantages, including minimal invasiveness, shorter anesthesia time, and a lower risk of intestinal dehiscence—factors particularly beneficial in dogs with poor general condition. The ability to obtain multiple samples from different gastrointestinal segments also increases diagnostic sensitivity. However, this technique has inherent limitations: focal lesions or abnormalities located beyond the reach of the endoscope may be missed, and because endoscopic biopsies are restricted to the mucosal layer, deeper submucosal or transmural lesions may not be adequately assessed [11].

Immunohistochemistry (IHC) and molecular clonality testing are valuable ancillary tools that can assist pathologists in diagnosing AL, particularly in cases where lymphoid morphology is ambiguous, such as with small-cell infiltrates. IHC provides phenotypic information by identifying the expression of specific lymphocyte markers (e.g., CD3 for T-cells, CD79a for B-cells), helping to characterize the lineage and distribution of infiltrating cells within the digestive system [2]. Clonality testing of lymphocyte populations detects gene rearrangements in T-cell receptor or immunoglobulin genes. A clonal population is suggestive of lymphoma, whereas a polyclonal pattern typically supports a diagnosis of non-neoplastic inflammatory enteropathy [12]. While these techniques are complementary and can improve diagnostic confidence, they are not interchangeable, as each has distinct strengths and limitations. Their combined use is particularly helpful in challenging cases where the histopathological findings are inconclusive.

The objective of this study was to characterize the clinical, biological, and endoscopic features of dogs with a diffuse form of AL, i.e., without an intestinal mass detectable upon abdominal ultrasound, at a veterinary referral center. We hypothesized that a diffuse form of AL is more prevalent in older dogs, presents with abnormal plasma albumin and serum cobalamin concentrations, has a poor prognosis without a specific treatment, and may present with recurrent endoscopic lesions.

## 2. Materials and Methods

### 2.1. Case Selection

All dogs with a histological diagnosis of AL at the referral hospital CHV OnlyVet (Saint-Priest, France), between December 2017 and November 2024, were retrospectively reviewed. Only dogs diagnosed with a diffuse form of the disease were included, providing that complete ultrasonographic, endoscopic, histological, and immunohistochemical reports were available. A diffuse form of AL was defined as a histological diagnosis of AL confirmed by immunohistochemical analysis, in the absence of a discernible mass upon abdominal ultrasound, with or without increased thickness and/or alterations in the structural layers of the gastrointestinal wall. Thickness of the intestinal walls was compared to the values in reference tables commonly used in veterinary gastrointestinal ultrasonography [13]. Regional mesenteric lymphadenopathy was defined as lymph nodes measuring >5 mm on ultrasonographic examination. Dogs with a well-defined intestinal mass or a suspicious but inconclusive histological diagnosis of AL were excluded. All dogs underwent a thorough examination of peripheral lymph nodes and were presumed to have primary AL in the absence of peripheral lymphadenopathy at the time of diagnosis. Dogs with concurrent neoplastic changes in extraintestinal organs in the abdominal cavity were also excluded. Additionally, dogs with primary lesions restricted to the anorectum were excluded due to the distinct nature and presumed favorable prognosis of ano-rectal lymphoma [14].

The dogs’ medical records were reviewed, and the data collected included breed; gender; age; clinical signs; and ultrasonographic, endoscopic and histological reports. The disease severity was evaluated by using the CCECAI (Canine Chronic Enteropathy Clinical Activity Index) scoring system [15]. The following variables were each scored on a scale from 0 to 3 according to established criteria [15]: attitude/activity, appetite, vomiting, stool consistency, stool frequency, weight loss, albumin levels, ascites and peripheral edema, and pruritus. The total CCECAI score was calculated by summing each value. Based on the total score, disease severity was categorized as follows: insignificant (0–3), mild (4–5), moderate (6–8), severe (9–11), and very severe (>12). The plasma albumin concentration was measured using the Catalyst One analyzer (IDEXX Laboratories, Westbrook, ME, USA; reference range, 23–40 g/L). Hypoalbuminemia was classified based on the CCECAI score as normal (>20 g/L), mild (15–19.9 g/L), moderate (12–14.9 g/L), or severe (<12 g/L). The serum cobalamin concentration was measured using IMMULITE 2000 XPi (Siemens, Munich, Germany, reference range, 234–812 ng/L). Although the reference laboratory defines the normal range for serum cobalamin as 234–812 ng/L, serum concentrations below 400 ng/L (suboptimal concentration, hypocobalaminemia or cobalamin deficiency) were considered abnormal in this study. This threshold was selected because serum cobalamin levels do not reliably reflect intracellular cobalamin status, and cobalamin supplementation is generally recommended in dogs when serum levels fall below 400 ng/L [16]. The survival time was defined as the interval between the date of endoscopic examination and the date of death. Follow-up information, including the date and cause of death, was obtained when available by contacting the referring veterinarians or the owners.

### 2.2. Sample Collection

Gastroduodenoscopy, ileocolonoscopy, or both were performed at the discretion of the primary clinician based on clinical assessment and abdominal ultrasound findings. All endoscopies were conducted using Olympus (Tokyo, Japan) GIF H-180, GIF Q-165, or GIF Q-180 equipment under general anesthesia by trained, board-certified internists (American or European College of Veterinary Internal Medicine, ACVIM-SAIM, or ECVIM-CA, respectively).

A minimum of 4 biopsies from each site (for gastroduodenoscopy: stomach and duodenum; for ileocolonoscopy: ileum and colon) were routinely collected using a 2.8 mm diameter Olympus biopsy forceps.

### 2.3. Endoscopic Evaluation

To reduce subjectivity in the assessment, all available endoscopic images for each case (at least one image per site) were independently and blindly reviewed by three board-certified internists (ACVIM-SAIM or ECVIM-CA). For gastroduodenoscopy, the evaluated sites included the esophagus, cardia, lesser and greater curvatures of the stomach, pylorus, and duodenum. For ileocolonoscopy, the colon and ileocolic sphincter were assessed. Video recordings were not available for review. An endoscopic examination report was completed for each case by each internist according to the criteria of the World Small Animal Veterinary Association (WSAVA) Gastrointestinal Standardization Group [17]. The assessed parameters included mucosal hyperemia/vascularity, edema, discoloration, friability, hemorrhage, erosions or ulcerations, mucosal texture, and lacteal dilatation. Due to the limitations of still images, some parameters—such as the presence of abnormal luminal content, sphincter function, and the ability to achieve adequate insufflation—could not be evaluated and were thus excluded from the endoscopic assessment. A standardized severity grading scale ranging from 0 to 3 was applied to each criterion (0 = absent, 1 = mild, 2 = moderate, and 3 = severe). For each criterion, the final score was calculated as the mean of the three individual assessments. The resulting average was then categorized as follows: 0 = absent, 0.5–1.5 = mild, 1.5–2.5 = moderate, and 2.5–3 = severe. On top of this standardized assessment, presence or absence of a “cobblestone” appearance of the duodenal mucosa was also noted for each case. The endoscopic “cobblestone” appearance refers to the presence of numerous elevated mucosal nodules or irregularities, giving the mucosal surface a lumpy, uneven texture. In human medicine, this pattern is classically associated with Crohn’s disease, but has also been reported in gastrointestinal infections such as *Cytomegalovirus* or *Yersinia* spp. [18].

### 2.4. Histopathology and Immunohistochemistry

Biopsies were fixed in 10% neutral buffered formalin. One of three board-certified pathologists from the European College of Veterinary Pathologists (ECVP), affiliated with the hospital at the time of diagnosis, performed the histological examination in accordance with WSAVA guidelines. Original reports were used for the purpose of this study. Lymphocyte epitheliotropism, heterogeneity, and nuclear size were routinely assessed [19]. Immunohistochemistry (IDEXX Laboratory, Saint-Denis, France) using CD3 antibodies (Dako, Glostrup, Denmark, A0452), CD79a antibodies (Bio-Rad, Hercules, CA, USA, MCA2538GA), and Ki67 (SP6) scores was performed on formalin-fixed samples from all dogs. The T-cell phenotype was determined by a predominant CD3-positive lymphoid population in the lamina propria and epithelium. The B-cell phenotype was based on CD79a positivity. The Ki67 score was determined by manually counting 500 cells on digitalized slides at 20× magnification. The percentage of positively stained nuclei among these 500 cells was recorded as the Ki67 score [20].

### 2.5. Statistical Analysis

Statistical analyses were performed using Jamovi software (version 2.5). Descriptive analyses focused on categorical variables and were reported as percentages. Data normality was assessed using Shapiro–Wilk tests. For normally distributed data, the results are presented as means (n; standard deviation (SD); and range). For non-normally distributed data, the results are reported as medians (n; interquartile range (IQR); and range).

Dogs with diffuse AL were divided in two groups according to the presence or absence of a “cobblestone” appearance of the duodenal mucosa. The comparison was performed as a post hoc analysis after all data had been collected. Ages, body weights, Ki67 values, plasma albumin concentrations, and CCECAI scores were compared between the two groups using Student’s *t*-test. Serum cobalamin concentrations were compared between the two groups by using the Mann–Whitney U test due to violation of variance homogeneity. Survival time was compared between the two groups by using the Kaplan–Meier test followed by log-rank analysis.

The concordance of the retrospective evaluation of endoscopic gastrointestinal lesions among the 3 board-certified internists was analyzed by using Kendall’s W correlation test. A strong association was defined as a Kendall’s tau-b value between 0.7 and 1, a moderate association as a value between 0.3 and 0.7, and a weak association as a value between 0 and 0.3.

The Kaplan–Meier product limit method was used to estimate survival. Survival rates at 1, 3, and 12 months were calculated.

A *p*-value < 0.05 was considered statistically significant.

## 3. Results

### 3.1. Signalment and Clinical Features (Table 1)

Thirty-nine dogs were recruited based on histological reports of alimentary lymphoma, including thirty dogs with a diffuse form of AL, eight dogs with a jejunal mass, and one dog with an esophageal mass. All nine dogs with a well-defined mass were excluded. Twelve dogs were further excluded due to a lack of complete histological and immunohistochemical analysis (*n* = 8) or because of incomplete endoscopic reports (*n* = 4). Eighteen dogs met the inclusion criteria.

The mean age was 7.81 years (*n* = 18; SD, 1.99; and range, 4–11). Seven dogs were intact males (39%), seven were spayed females (39%), three were neutered males (17%), and one was an intact female (5%). The mean body weight was 19.3 kg (*n* = 18; SD, 10.3; and range, 4.2–35.6). The represented breeds were Pugs (*n* = 3), French bulldogs (*n* = 2), and a minority of other pure breeds (one dog per breed, *n* = 13). All of the dogs were presented for the investigation of chronic gastrointestinal signs. Fifteen dogs had weight loss (83%), fourteen dogs had diarrhea (78%), twelve dogs had vomiting (67%), and five dogs showed signs of gastrointestinal bleeding (28%). The CCECAI scores were assessed in 17 dogs and were insignificant in none of the dogs, mild in 1 dog (6%), moderate in 4 dogs (24%), severe in 7 dogs (41%), and very severe in 5 dogs (29%). The mean CCECAI score was 9.65 (*n* = 17; SD, 2.42; and range, 5–13).

**Figure 1:**
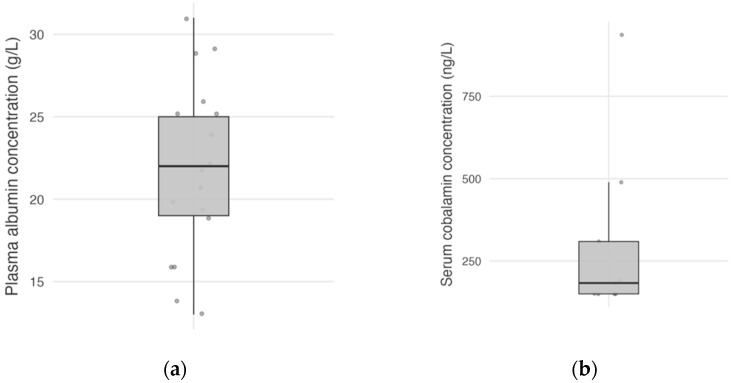
Distribution of plasma albumin concentrations in 17 dogs (**a**), and serum cobalamin concentrations in 9 dogs (**b**). Each point represents a dog.

Plasma albumin concentration was available for 17 dogs (94%), with a mean plasma albumin concentration at the time of diagnosis of 21.8 g/L (*n* = 17; SD, 5.34; and range, 13–31, Figure 1a). Plasma albumin concentration was normal in 11 dogs (65%), mildly decreased in 4 dogs (23%), and moderately decreased in 2 dogs (12%), and none of the dogs had severe hypoalbuminemia. Serum cobalamin concentration was available in nine dogs (50%), with a median serum cobalamin concentration of 183 ng/L (*n* = 9; IQR, 150–309; and range, <150–937; Figure 1b).

### 3.2. Diagnostic Imaging Findings

The main ultrasonographic findings included paralytic ileus with corrugated intestinal loops (72%), and heterogeneous and hyperechoic intestinal walls (56%). Intestinal wall thickening was identified in the stomachs of five dogs (28%), in the duodenums of eight dogs (44%), in the jejunums of five dogs (28%), in the ileums of five dogs (28%), and in the colons of four dogs (22%). In three cases (17%), a segmental loss of normal wall layering was observed without a clearly defined mass. Regional mesenteric lymphadenopathy was present in eight dogs (44%; range: 5.2–22.5 mm).

### 3.3. Endoscopic Findings

Ten dogs (10/18, 56%) had a combination of gastroduodenoscopy and ileocolonoscopy, seven dogs (39%) had gastroduodenoscopy alone, and one dog (5%) had ileocolonoscopy alone. Concordance between the three internist clinicians upon reviewing the endoscopic images was considered moderate on the basis of Kendall W correlation and was similar between the three specialists (Kendall’s tau-b ranging from 0.534 to 0.582). The highest agreement was observed between clinicians 2 and 3, with a tau-b of 0.582 (*p* < 0.001). The results of the correlations are shown in Table 2.

**Figure 2:**
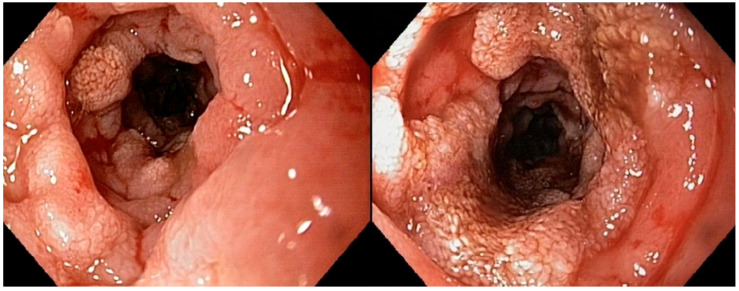
Duodenoscopy of dog n°9 (confirmed large-T-cell duodenal lymphoma): very severe alteration in the granularity of the duodenal mucosa.

**Figure 3:**
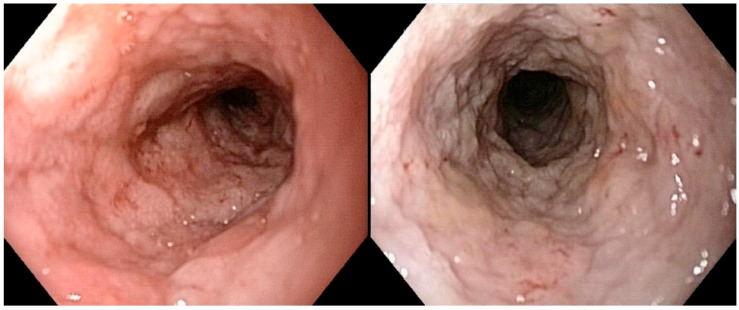
Duodenoscopy of dog n°6 (confirmed large-T-cell duodenal lymphoma): “cobblestone” appearance of the duodenal mucosa.

All of the endoscopic findings are summarized in Table 3. Figure 2 illustrates a severe alteration in the granularity of the duodenal mucosa during duodenoscopy. Duodenal lacteal dilatation was observed in one dog (6%). A “cobblestone” appearance of the duodenal mucosa was identified in nine dogs (53%) (Figure 3). No dog developed colonic stricture or intussusception.

### 3.4. Histological Findings

**Figure 4:**
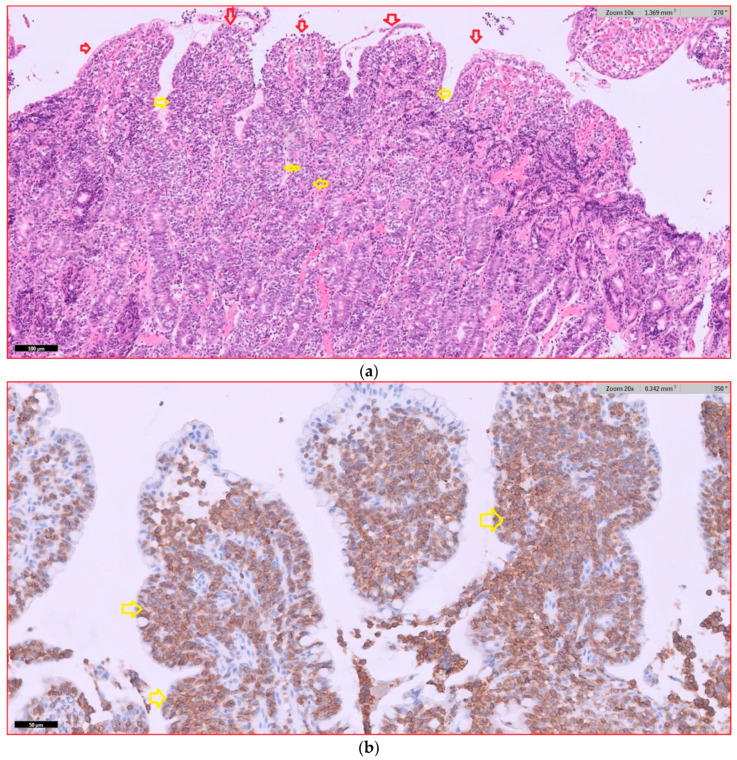
(**a**) Intermediate- to large-T-cell duodenal lymphoma, hematoxylin and eosin (HE). Villous atrophy and broadening are visible (red arrows). Neoplastic lymphocytes show epitheliotropism within villi and crypt epithelium (yellow arrows). (**b**) Intermediate- to large-T-cell duodenal lymphoma, CD3 immunohistochemistry (Dako, A0452). Marked predominance of CD3-positive lymphoid cells is observed, with pronounced epitheliotropism (yellow arrows).

In dogs with diffuse AL that underwent endoscopic examination, 17 dogs had intermediate- to large-cell lymphoma (94%), and 1 dog (6%) had small- to intermediate-cell lymphoma. The duodenum was the part of the gastrointestinal tract most frequently affected by lymphoma. Infiltration by lymphoma was observed in the duodenums of 15 dogs, in the ileums of 5 dogs, in the stomachs of 3 dogs, and in the colons of 2 dogs. Six dogs had lymphomatous infiltration in multiple locations simultaneously. Dogs with duodenal lymphoma exhibited nests of neoplastic cells (80%, 12/15), villous atrophy (60%, 9/15), marked epitheliotropism (60%, 9/15), crypt distension (40%, 6/15), and lacteal dilatation (7%, 1/15). Dogs with ileal lymphoma showed nests of neoplastic cells (100%, 5/5), villous atrophy (80%, 4/5), marked epitheliotropism (80%, 4/5), and crypt distension (40%, 2/5), while no cases of lacteal dilatation were observed. Representative histopathological images of intermediate- to large-T-cell duodenal lymphoma are shown in Figure 4.

### 3.5. Immunohistochemistry Findings (Table 4)

Sixteen dogs (16/18; 89%) exhibited a T-cell phenotype, while only two dogs (2/18; 11%) showed a B-cell phenotype. The Ki67 score was obtained for all dogs. The mean Ki67 score was 11.7% (*n* = 18; SD, 5.23; and range, 3.6–26). The dog with small-cell AL had a Ki67 score of 14.5%.

### 3.6. Relationship Between Biological Data and Endoscopic and Histological Findings

In the only dog with gastric and duodenal small-cell lymphoma, the endoscopic lesions of the stomach were all classified as ‘moderate’ for mucosal hyperemia, edema, discoloration, and the presence of mucosal ulcerations. The duodenal mucosa of this dog showed severely altered granularity, with no ulcerations or a “cobblestone” appearance.

**Figure 5:**
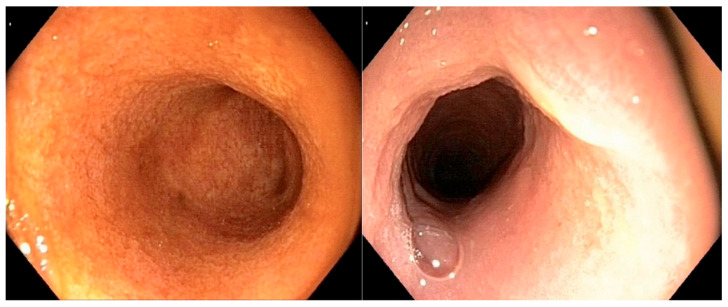
Duodenoscopy of dog n°11 (confirmed large-T-cell duodenal lymphoma). Nearly normal appearance of the duodenal mucosa, which is mildly hyperemic (left picture), with no significant ulcerations or granularity changes despite the histological diagnosis of duodenal large-T-cell lymphoma.

In dogs with intermediate- to large-cell duodenal lymphoma (*n* = 14), none had a normal duodenal mucosa. The alteration in duodenal granularity was reported to be severe in six dogs (43%), moderate in five dogs (36%), and mild in three dogs (21%). Duodenal ulcerations were absent, mild, and moderate in two (14%), eight (57%), and four (29%) dogs, respectively; no dog had severe duodenal ulceration. Duodenal lacteal dilatation was present in only one dog with duodenal lymphoma (moderate- to large-cell). Three dogs (cases 1, 11, and 18) with duodenal intermediate- to large-cell lymphoma showed only mild changes (of any type) in the duodenal mucosa (Figure 5).

**Figure 6:**
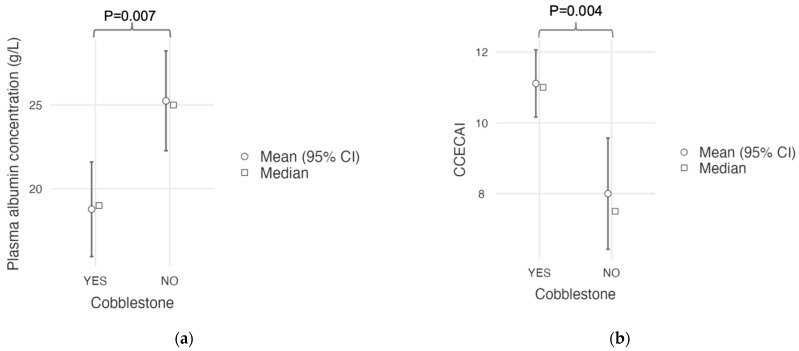
(**a**) Plasma albumin concentrations and (**b**) CCECAI scores in dogs with diffuse alimentary lymphoma with (YES) or without (NO) a “cobblestone” appearance of the duodenal mucosa; Student‘s *t*-test.

A “cobblestone” appearance of the duodenal mucosa was identified in nine dogs with duodenal lymphoma (9/15; 60%) and in neither of the two dogs without duodenal involvement who underwent gastroduodenoscopy. All nine of these dogs (100%) with a “cobblestone” appearance of the duodenal mucosa had confirmed duodenal involvement from intermediate- to large-cell lymphoma. A comparative analysis of clinical and histological quantitative parameters between dogs with and without this endoscopic feature is shown in Table 5. Dogs with a “cobblestone” appearance of the duodenal mucosa showed a significantly lower plasma albumin concentration (mean, 18.8 g/L; SD, 4.32; and range, 13–26) compared to dogs without a “cobblestone” appearance (mean, 25.3 g/L; SD, 4.3; and range, 19–31; *p* = 0.007; Figure 6a). They also had a significantly higher CCECAI score (mean, 11.1; SD, 1.45; and range, 9–13) than dogs without a “cobblestone” appearance (mean, 8.0; SD, 2.27; and range, 5–12. *p* = 0.004; Figure 6b). Age, body weight, serum cobalamin concentration, and Ki67 score did not differ between groups (Table 5).

### 3.7. Treatments and Outcomes

After endoscopic examination, all dogs received prednisolone at variable doses (0.7–1.3 mg/kg/day), and six dogs (33%) received antibiotics, primarily metronidazole (*n* = 3) followed by marbofloxacine (*n* = 2) and amoxicillin-clavulanate (*n* = 1). After histopathological confirmation of the diagnosis, maximal-tolerated-dose chemotherapy protocols (CHOP protocols) were proposed for dogs still alive at the time; however, none of the owners elected to pursue this treatment option. Three dogs received a combination of prednisolone (1 mg/kg/day) and chlorambucil (4 mg/m^2^ q24–48 h), resulting in survival times of 9 days, 58 days, and 349 days, respectively. These treatments were not part of the standardized chemotherapy protocol and were administered at the clinician’s discretion.

At the time of writing, 16 dogs (89%) have died, all because of AL. Two dogs (11%) were still alive at 170 (case 13) and 563 days (case 17) since diagnosis; these two dogs were censored for the survival analysis. The median survival time was 30.5 days (*n* = 16; IQR, 9–84.5; and range, 4–350). Among the dogs that finally died, eight, four, and zero dogs were still alive at 1, 3, and 12 months after diagnosis, respectively.

**Figure 7:**
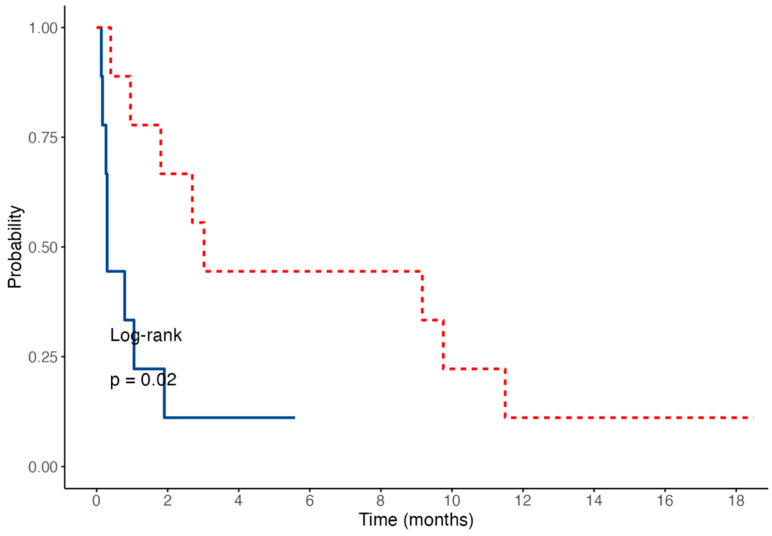
A Kaplan–Meier survival curve of dogs with a diffuse form of alimentary lymphoma, comparing survival based on the presence (solid blue line) or absence (dashed red line) of a cobblestone appearance of the duodenal mucosa.

The median survival time was significantly shorter in dogs with a “cobblestone” appearance of the duodenal mucosa (median: 9 days; range, 4–58) compared to those without one (median: 92 days; range, 12–350; log-rank test: *p* = 0.02; Figure 7).

## 4. Discussion

Diffuse AL can be more challenging to diagnose than mass-associated forms, due to its clinical resemblance to severe chronic enteropathies. Our study aims to describe the clinical, biological, endoscopic, histological, and immunohistochemical findings in dogs with diffuse AL. In the present study, dogs diagnosed with diffuse AL were generally middle-aged or older, the youngest dog being 4 years old. An earlier study reported that males had a predisposition for developing high-grade AL, but no sex predilection was identified in our study [21]. In a previous study of canine transmural lymphoma [3], Pug dogs were overrepresented, accounting for 17% of T-cell AL cases. Similarly, in our study, the Pug was the most commonly affected breed (17%).

Plasma albumin concentration is a key prognostic biomarker in protein-losing enteropathies, with one study reporting that normalization within 50 days of treatment initiation was associated with a longer survival time [22]. Surprisingly, in our study, 65% of dogs had normal plasma albumin concentrations according to the CCECAI scoring system, despite the severity of endoscopic lesions observed in most cases and the presence of duodenal or ileal infiltration in all but one dog. This discrepancy may be explained by the segmental nature of the lymphoproliferative infiltrate, which may spare large portions of the intestinal mucosa and thus have a limited impact on nutrient absorption. Alternatively, albumin levels may have been overestimated in some dogs due to dehydration secondary to gastrointestinal losses. The prognostic value of hypoalbuminemia in diffuse AL should be evaluated in future studies including larger cohorts of dogs with a severe degree of hypoalbuminemia.

In dogs with chronic enteropathies, low serum cobalamin levels (<200 ng/L) were significantly associated with poorer outcomes, despite 6 weeks of cobalamin supplementation [15]. In our study, serum cobalamin concentrations were available for nine dogs, with a median serum cobalamin concentration of 183 ng/L (*n* = 9; IQR, 150–309; and range, <150–937). Seven dogs had a serum cobalamin concentration <400 ng/L, including three dogs with concentrations <150 ng/L. Serum cobalamin concentrations < 150 ng/L could not be precisely measured by the reference laboratory due to the extremely low concentrations. Of the seven dogs with cobalamin levels lower than 400 ng/L, involvement of the ileum was reported in only two dogs. However, three out of seven dogs did not undergo ileocolonoscopy; therefore, ileal involvement may have been underestimated. Considering the high proportion of dogs with hypocobalaminemia in our study, the routine assessment of serum cobalamin in cases of diffuse AL appears warranted, even though its prognostic value was not determined in the present cohort.

In the literature, the most commonly reported ultrasonographic findings of AL in dogs include a thickened gastric or intestinal wall, a loss of layering, the presence of a mass, or ulceration [4]. The ultrasonographic results in our study revealed that the duodenum was the most frequently affected segment of the gastrointestinal tract. This finding aligns with the histological predominance of lymphomatous infiltration observed primarily in the duodenums of affected dogs. However, the absence of duodenal wall thickening does not exclude the presence of duodenal lymphoma.

Human gastroenterologists use endoscopic features to help differentiate aggressive from non-aggressive AL. Mucosal lesions are classified according to their appearance: the superficial type, protruding type without ulceration, protruding type with ulceration, fungating type, multiple-nodule type, and giant fold type [10]. Although no endoscopic lesion is pathognomonic for the type of AL, human gastroenterologists identify certain lesions, such as fungating lesions and protruding lesions with ulceration, as being significantly associated with aggressive non-Hodgkin’s lymphoma [10]. Although a standardized approach to gastrointestinal endoscopic evaluation has been proposed in veterinary medicine by the WSAVA Gastrointestinal Standardization Group, no specific consensus exists for the assessment of digestive neoplasms. Consequently, the WSAVA scoring system is primarily intended to ensure a systemic evaluation of each accessible segment of the gastrointestinal tract, rather than to provide a detailed characterization of mucosal abnormalities. This contrasts with human gastroenterology, where mucosal changes are more rigorously assessed when a suspicious lesion is identified.

One of our main aims was to provide an endoscopic description of lesions observed with diffuse AL. To the authors’ knowledge, this is the second study to describe the endoscopic features of AL in dogs, and the first to specifically document endoscopic findings in cases with a diffuse form of the disease. The only previously published study included seven dogs with AL, four of which exhibited a “cobblestone” appearance of the duodenal mucosa, consistent with histologically confirmed duodenal infiltration [23]. Our findings expand upon these preliminary observations by focusing on the diffuse form of the disease and systematically correlating endoscopic features with histopathological and immunohistochemical data. In our study, the duodenum was the most frequently affected segment of the gastrointestinal tract, with lymphomatous infiltration of the mucosa observed in 15 out of 17 dogs (83%). Altered mucosal granularity was the most commonly reported abnormality and was present in all dogs with duodenal lymphoma. Severe changes in granularity were reported in 41% (7/17) of dogs, one of which had a small- to intermediate-cell duodenal lymphoma. A “cobblestone” appearance of the duodenal mucosa was noted in 9 out of 17 dogs (53%) and was significantly associated with lower plasma albumin concentrations and higher CCECAI scores. All affected dogs were diagnosed with intermediate- to large-cell duodenal lymphoma. Therefore, a “cobblestone” pattern in the duodenum should be considered a potential indicator of disease severity. This endoscopic feature has already been reported in dogs with duodenal lymphoma, leading authors to propose that AL should be strongly suspected when a solitary mass or a cobblestone appearance is observed on endoscopic examination [23]. However, because our study did not include dogs with chronic inflammatory enteropathies, we cannot determine whether this feature is specific to lymphoma. A comparative study including both healthy control dogs and dogs with non-neoplastic chronic enteropathies would be required to assess the diagnostic relevance of this pattern. Furthermore, due to the limited number of cases with gastric, ileal, or colonic lymphoma in our study, no firm conclusions can be drawn regarding the endoscopic appearance of these segments.

Endoscopic gastrointestinal biopsies appear to be an appropriate sampling method with which to diagnose lymphoma, since 75% of ALs are epitheliotropic [2]. This minimally invasive technique allows samples to be taken quickly while reducing anesthetic time and decreasing the risk of intestinal dehiscence, particularly in cases in poor general condition and with hypoalbuminemia. An additional advantage of endoscopy is the ability to obtain multiple biopsies from various sites along the gastrointestinal tract, thereby increasing the diagnostic yield. In contrast, surgical biopsies obtained via laparotomy typically involve only a limited number of intestinal segments, although they allow for full-thickness samples and the assessment of all layers of the intestinal wall. Nevertheless, endoscopy has inherent limitations: focal lesions or those located beyond the reach of the endoscope may be missed, and lesions confined to the submucosa or deeper layers may not be detected through mucosal biopsies alone. These factors may contribute to the underdiagnosis of lymphomas that do not exhibit epitheliotropism when relying exclusively on endoscopic sampling [2].

There are notable species-specific differences in the forms of AL. In humans, the most common subtype of AL is B-cell non-Hodgkin’s lymphoma, particularly diffuse large-B-cell lymphoma (DLBCL) and mucosa-associated lymphoid tissue (MALT) lymphoma [24]. Conversely, T-cell lymphomas are rare in humans, accounting for only 4–6% of AL cases [24]. According to our study, T-cell AL appears to be the most common form of diffuse AL in dogs, underlining a significant divergence in lymphoma subtypes between species.

Diffuse AL in our study was associated with a poor prognosis, resulting in a median survival time of 30.5 days (*n* = 16; IQR, 9–84.5; and range, 4–350). The presence of a duodenal mucosa with a “cobblestone” appearance appeared to be associated with a worse outcome: the median survival time was significantly shorter in dogs with this endoscopic feature (median: 9 days; range, 4–58) compared to those without (median: 92 days; range, 12–350; *p* = 0.02). The results of our study highlight the aggressive nature of diffuse AL, particularly in the absence of an appropriate chemotherapeutic protocol. These findings are consistent with previous reports of similar outcomes, particularly in cases of intestinal large-cell T lymphoma, for which a median survival time of 62 days has been reported [5]. Slightly better outcomes were obtained in some studies, particularly when dogs received a multi-drug chemotherapy protocol [4,25]. In our study, none of the dogs received a maximum-tolerated-dose chemotherapy protocol. This may be attributed to the rapid deterioration of the clinical condition of many dogs before their histopathological results became available. Additionally, the cost of a chemotherapy protocol combined with the guarded prognosis may have discouraged owners from pursuing treatment. Further studies are required to evaluate the prognosis of dogs with diffuse AL undergoing polychemotherapy.

The majority of previous studies describing AL focused on immunohistochemical and molecular findings [3,6,19,20,26]. Canine intestinal lymphoma is morphologically classified into small-cell, intermediate-cell, or large-cell lymphoma according to its nuclear size [3]. Based on our histological results, most of our dogs (94%) had intermediate- to large-cell AL. IHC carried out on all dogs confirmed the predominance of the T-cell phenotype (89%) associated with canine AL, in accordance with the literature [3,5,6,27,28]. In the B-cell phenotype, one dog had a large-B-cell gastric lymphoma and the other one had a large-B-cell ileo-colic lymphoma. Based on these results, it is not possible to determine whether the phenotype could have an impact on the expected survival of these dogs. The two dogs with B-cell AL survived for 279 days and 29 days, but we did not identify any potential factors that might have influenced these widely divergent results. The Ki67 score is a marker of cellular proliferation used in both canine and human lymphoma to correlate cellular proliferation with prognosis [19]. Although some studies have specifically focused on the use of the Ki67 score to differentiate canine inflammatory bowel disease (IBD) from intestinal lymphoma, it appears that the Ki67 score cannot reliably distinguish between IBD and lymphoma [19]. In the aforementioned study, lymphomas exhibited significantly higher Ki67 scores (30–62%) compared to inflammatory enteropathy cases, where most showed indices below 25% [19]. Surprisingly, the mean Ki67 score in our study was much lower; however, the histopathological findings of our dogs with intermediate- to large-cell lymphoma did not suggest that the final diagnosis was questionable. This further emphasizes that the Ki67 score should not be used as the sole tool for diagnosing and prognosticating AL.

The limitations of this study are mainly due to its retrospective nature, including the absence of biochemical analysis results in all cases, particularly serum cobalamin and plasma albumin concentrations, and inconsistent chemotherapeutic protocols. The relatively small number of included cases is also a limitation, as it may have reduced the statistical power of the study. This limited sample size is mainly attributable to the stringent inclusion criteria, which required histopathological and immunohistochemical confirmation and therefore excluded cases diagnosed by cytological analysis alone. This study does not allow us to report the overall prevalence of dogs with AL in a referral clinic, as many cases of AL were diagnosed by fine-needle aspiration during ultrasound examination of the abdomen. This selection bias excluded all dogs with intestinal masses for which surgical management was recommended in the event of an intestinal obstruction. The absence of a control group including both healthy dogs and dogs with non-neoplastic chronic enteropathies prevents any assessment of whether the reported endoscopic lesions are specific to AL. Moreover, the concordance between the descriptions of gastrointestinal lesions by the three board-certified internists was only moderate, based on the Kendall W correlation. This highlights the relative subjectivity of endoscopic observations despite well-defined criteria, and the need to perform systematic multiple biopsies, even if the lesions appear to be of minor significance to the operator, to optimize the chances of obtaining a reliable diagnosis. As a result, the sensitivity and specificity of these endoscopic findings for the diagnosis of AL could not be determined. Further prospective studies with well-defined control groups are needed to clarify the diagnostic value of such endoscopic features. Additionally, clonality assessment via PARR (polymerase chain reaction for antigen receptor rearrangements) was not routinely performed in this study, especially for cases of small- to intermediate-cell lymphoma. This decision was mainly influenced by logistical constraints, including cost and accessibility. Finally, the relatively small cohort restricts the statistical power of our results.

## 5. Conclusions

In conclusion, this study emphasizes the critical importance of the early recognition of diffuse AL, as the condition carries a poor prognosis, especially when not treated with appropriate chemotherapy. Although the mass-forming presentation of AL is more commonly observed in dogs, the diffuse form may not be as rare as previously thought and should be considered as a differential diagnosis in dogs presenting with signs of chronic enteropathy. Moreover, diffuse forms in the gastrointestinal tract should be suspected, even in the absence of significant ultrasound abnormalities or endoscopic lesions. Although certain endoscopic lesions, particularly a “cobblestone” appearance of the duodenal mucosa, were significantly associated with lower plasma albumin concentrations and higher CCECAI scores, the subtle nature of endoscopic lesions in some dogs underscores the need for further investigation. In dogs with chronic gastrointestinal signs and biological findings suggestive of severe enteropathy, the authors recommend at least performing endoscopic gastrointestinal biopsies in all cases to differentiate diffuse AL from severe chronic enteropathy. Further prospective studies would be valuable to provide more-comprehensive data on the prognosis of canine AL, and to guide treatment strategies, notably chemotherapy protocols.

## Figures and Tables

**Table 1:** Signalment and clinical data of 18 dogs with a diffuse form of alimentary lymphoma. Abbreviations: NM, neutered male; IM, intact male; SF, spayed female; IF, intact female; CCECAI, Canine Chronic Enteropathy Clinical Activity Index; and NA, not available.

Case Number	Gender	Age (Years)	Lymphoma Location	CCECAI Score	Survival Time (Days)
1	NM	9	Stomach and duodenum	5	297
2	SF	11	Duodenum and ileum	9	8
3	SF	8	Stomach	8	279
4	SF	8	Duodenum, ileum, and colon	11	24
5	SF	6	Duodenum	10	58
6	SF	6	Duodenum and ileum	12	9
7	IM	8	Duodenum	10	82
8	SF	10	Duodenum	12	9
9	NM	4.5	Duodenum	13	5
10	SF	9	Duodenum	6	350
11	IM	4	Duodenum	9	12
12	IM	10	Duodenum	10	4
13	IM	9	Duodenum	13	Still alive
14	IM	7	Ileum	7	55
15	IM	9	Ileum and colon	7	29
16	IM	5	Duodenum	10	32
17	IF	9	Stomach and duodenum	12	Still alive
18	NM	8	Duodenum	NA	92

**Table 2:** Summary table of the Kendall W correlation test between the three internist clinicians who evaluated the 18 cases of gastrointestinal endoscopy. A strong association is defined by a Kendall’s tau-b value between 0.7 and 1, a moderate association as a value between 0.3 and 0.7, and a weak association as a value between 0 and 0.3.

		Clinician 1	Clinician 2	Clinician 3
Clinician 1	Kendall tau-B *p*-value	- -		
Clinician 2	Kendall tau-B *p*-value	0.566 <0.001	- -	
Clinician 3	Kendall tau-B *p*-value	0.534 <0.001	0.582 <0.001	- -

**Table 3:** Endoscopic findings in 18 dogs: 10 dogs underwent both gastroduodenoscopy and ileocolonoscopy, 7 dogs underwent gastroduodenoscopy only, and 1 dog underwent ileocolonoscopy only.

	Severity *n* (%)			
Esophagus (*n* = 17)	Absent	Mild	Moderate	Severe
Esophagitis	17 (100)	0	0	0
Hyperemia	12 (71)	5 (29)	0	0
Discoloration	16 (94)	1 (6)	0	0
Erosion/ulceration	16 (94)	1 (6)	0	0
Dilatation	16 (94)	1 (6)	0	0
Stomach (*n* = 17)				
Hyperemia	3 (18)	9 (53)	5 (29)	0
Edema	5 (29)	6 (36)	5 (29)	1 (6)
Discoloration	5 (29)	10 (59)	2 (12)	0
Erosion/ulceration	10 (59)	3 (18)	4 (23)	0
Duodenum (*n* = 17)				
Hyperemia	2 (12)	8 (47)	4 (23)	3 (18)
Edema	2 (12)	6 (36)	7 (41)	2 (12)
Granularity	0	3 (18)	7 (41)	7 (41)
Erosion/ulceration	4 (23)	9 (53)	4 (23)	0
Colon (*n* = 11)				
Hyperemia	3 (28)	4 (36)	4 (36)	0
Discoloration	4 (36)	6 (54)	1 (10)	0
Erosion/ulceration	7 (64)	2 (18)	2 (18)	0

**Table 4:** Alimentary lymphoma in 18 dogs: location, cell size, immunophenotype, and Ki67 score. Abbreviations: GD, gastroduodenoscopy; IC, ileocolonoscopy; +, positive; and −, negative.

Case Number	Endoscopic Evaluation	Lymphoma Location	Cell Sizes	CD3	CD79a	Ki67 (%)
1	GD	Stomach and duodenum	Intermediate to large	+	−	11.6
2	GD and IC	Duodenum and ileum	Intermediate to large	+	−	10.6
3	GD	Stomach	Intermediate to large	−	+	8
4	GD and IC	Duodenum, ileum, and colon	Intermediate to large	+	−	7.2
5	GD	Duodenum	Intermediate to large	+	−	11
6	GD and IC	Duodenum and ileum	Intermediate to large	+	−	17.8
7	GD	Duodenum	Intermediate to large	+	−	12.6
8	GD and IC	Duodenum	Intermediate to large	+	−	18.2
9	GD	Duodenum	Intermediate to large	+	−	13
10	GD and IC	Duodenum	Intermediate to large	+	−	5.6
11	GD and IC	Duodenum	Intermediate to large	+	−	3.6
12	GD and IC	Duodenum	Intermediate to large	+	−	14.6
13	GD and IC	Duodenum	Intermediate to large	+	−	26
14	GD and IC	Ileum	Intermediate to large	+	−	7.8
15	IC	Ileum and colon	Intermediate to large	−	+	12.2
16	GD and IC	Duodenum	Intermediate to large	+	−	6.9
17	GD	Stomach and duodenum	Small to intermediate	+	−	14.5
18	GD	Duodenum	Intermediate to large	+	−	12.2

**Table 5:** Association between the presence (YES) or absence (NO) of a “cobblestone” appearance of the duodenal mucosa and clinical and histological numerical variables in dogs with a diffuse form of alimentary lymphoma.

Variables	Cobblestone	*n*	Mean	SD	*p*-Value
Age (years)	YES	9	7.72	2.41	0.865
	NO	9	7.89	1.62	
Body weight (kilograms)	YES	9	15.18	11.63	0.09
	NO	9	23.40	7.13	
CCECAI	YES	9	11.11	1.45	0.004 *
	NO	8	8.00	2.27	
Ki67 (%)	YES	9	13.77	5.97	0.09
	NO	9	9.59	3.57	
Plasma albumin concentration (g/L)	YES	9	18.78	4.32	0.007 *
	NO	8	25.25	4.30	

Statistical differences (Student’s *t*-test) are indicated by an asterisk (*). Abbreviations: CCECAI, Canine Chronic Enteropathy Clinical Activity Index; *n*, number; and SD, standard deviation.

## Data Availability

The data is contained within the article.

## Abbreviations

The following abbreviations are used in this manuscript:

ACVIM-SAIM	American College of Veterinary Internal Medicine—Small Animal Internal Medicine
AL	Alimentary Lymphoma
CCECAI	Canine Chronic Enteropathy Clinical Activity Index
DLBCL	Diffuse Large-B-cell Lymphoma
ECVIM-CA	European College of Veterinary Internal Medicine—Companion Animals
IBD	Inflammatory Bowel Disease
IHC	Immunohistochemistry
LGITL	Low-Grade Intestinal T-cell Lymphoma
MALT	Mucosa-Associated Lymphoid Tissue lymphoma
PARR	Polymerase Chain Reaction for Antigen Receptor Rearrangement
WHO	World Health Organization
WSAVA	World Small Animal Veterinary Association
